# Effects of Dilution Systems in Olfactometry on the Recovery of Typical Livestock Odorants Determined by PTR-MS

**DOI:** 10.3390/s17081859

**Published:** 2017-08-11

**Authors:** Pernille Lund Kasper, Dietmar Mannebeck, Arne Oxbøl, Jens Vinge Nygaard, Michael Jørgen Hansen, Anders Feilberg

**Affiliations:** 1 Department of Engineering, Aarhus University, Hangøvej 2, DK-8200 Aarhus N, Denmark; peka@eng.au.dk (P.L.K.); jvn@eng.au.dk (J.V.N.); michaelj.hansen@eng.au.dk (M.J.H.); 2 Olfasense GmbH, Fraunhoferstraße 13, DE-24118 Kiel, Germany; dmannebeck@olfasense.com; 3 FORCE Technology, Park Allé 345, DK-2605 Brøndby, Denmark; aox@force.dk

**Keywords:** dynamic olfactometry, EN13725, olfactometer, PTR-MS, odor, *n*-butanol

## Abstract

The present study provides an elaborate assessment of the performance of olfactometers in terms of odorant recovery for a selection of odorants emitted from livestock houses. The study includes three different olfactometer dilution systems, which have been in use at accredited odor laboratories. They consist of: (i) a custom-built olfactometer made of glass tubes, (ii) a TO8 olfactometer, and (iii) an Olfacton dilution system based on a mass flow controller. The odorants include hydrogen sulfide, methanethiol, dimethyl sulfide, acetic acid, butanoic acid, propanoic acid, 3-methylbutanoic acid, 4-methylphenol, and trimethylamine. Furthermore, *n*-butanol, as the reference gas in the European standard for olfactometry, EN13725, was included. All measurements were performed in real time with proton-transfer-reaction mass spectrometry (PTR-MS). The results show that only dimethyl sulfide was almost completely recovered in all cases, while for the remaining compounds, the performance was found to vary significantly (from 0 to 100%) depending on the chemical properties of the compounds, the concentration levels, the pulse duration, and the olfactometer material. To elucidate the latter, the recovery in different locations of the TO8 olfactometer and in tubes of different materials, that is, poly-tetrafluoroethylene (PTFE), perfluoroalkoxy (PFA), stainless steel and SilcoTek-coated steel, were tested. Significant saturation effects were observed when odorants were in contact with stainless steel.

## 1. Introduction

To measure odor at animal production facilities, it is common practice to collect air samples at the source, store them in sample bags for up to 30 h, and subsequently dilute them in an olfactometer to assess them with human panelists. This sample procedure is typically performed according to a standard such as the European standard for dynamic olfactometry, EN13725 [1]. However, a major challenge, which needs to be considered when applying this method, is the odorant’s adsorption to the inner surfaces of the sampling equipment, for example, sample bags and olfactometers. The European standard for dynamic olfactometry [1] defines the permissible materials for the construction of sampling equipment to minimize the sample contamination and/or alteration. According to the standard, the materials in an olfactometer, which are directly exposed to the sample, should be made of one of poly-tetrafluoroethylene (PTFE)/Teflon, FEP, PET, glass or stainless steel.

Several studies have documented losses of odorants through adsorption and/or diffusion in different types of sample bags, and the chemical composition of samples containing odorants from livestock production facilities has been shown to alter significantly during storage [2,3,4,5]. On the other hand, the effect on the chemical composition of samples, when exposed to the materials in an olfactometer, has been investigated to a much lesser extent. Hansen et al. [6] found that 10–60% of reduced sulfur compounds, that is, hydrogen sulfide, methanethiol and dimethyl sulfide, were not recovered when they were diluted in two types of olfactometers constructed of glass and stainless steel/PTFE, respectively. In another study by Hansen et al. [7], it was shown that reduced sulfur compounds (hydrogen sulfide and methanethiol), *n*-butanol, carboxylic acids, 3-methylphenol and trimethylamine were not fully recovered in a TO8 olfactometer, and for the latter three, the pulse duration (i.e., the period of time for which the panelists are presented to the sample) was shown to have a large impact on the chemical outcome of a measurement. A similar dependence on the pulse duration for hydrogen sulfide was found by Beauchamp et al. [8].

Taking into account the very limited research published about the adsorption/desorption effects on odor samples in olfactometers and the reported discrepancies between measurements performed at different odor laboratories [9,10], performed with different olfactometers [11,12], and performed with a high variability in odor threshold values [13,14,15], there is a need to clarify the practical significance of the losses occurring in different types of olfactometers. This is necessary to validate the method and to test the credibility of the measurements. Furthermore, the fate of the compounds lost should be determined if these issues are to be minimized. Because the concentration levels of odorous compounds in environmental sample matrices, for example, samples collected at animal production facilities, are typically in the low ppb_v_ range, it is plausible that losses occurring during olfactometric measurements could have a significant impact on the results of odor analysis. This could cause an underestimation of the true odor concentration or a misinterpretation when evaluating odor-abatement technologies.

Hence, the objective of this work is to evaluate how different olfactometers and different materials may influence odor samples during dynamic olfactometry. This was accomplished by testing the time-resolved recovery of a series of key odorants from pig production [16,17,18] in three different olfactometer dilution systems made of different materials and with different configurations, although all were confined within the European standard. Furthermore, additional material tests were carried out. Measurements were performed with proton-transfer-reaction mass spectrometry (PTR-MS) with a high time resolution allowing for real-time measurements of the concentration profiles during dilution in the olfactometers.

## 2. Materials and Methods

### 2.1. Odorants

The odorous compounds included in the study were reduced sulfur compounds (hydrogen sulfide, methanethiol, and dimethyl sulfide), carboxylic acids (acetic acid, butanoic acid, propanoic acid, and 3-methylbutanoic acid), 4-methylphenol (p-cresol), trimethylamine, and *n*-butanol. The odorants were introduced into the olfactometers as single compounds. Certified *n*-butanol (AGA, Copenhagen, Denmark), hydrogen sulfide, methanethiol, and dimethyl sulfide (Air Liquide, Horsens, Denmark) were introduced from standardized gas cylinders. Acetic acid, butanoic acid, propanoic acid, 3-methylbutanoic acid and 4-methylphenol were generated from permeation tubes (VICI Metronics, Inc., Houston, TX, USA) using a permeation oven (Dynacalibrator model 150, VICI Metronics Inc., Houston, TX, USA). Trimethylamine was supplied from a permeation tube made of Teflon with brass fittings and a liquid solution of 45 wt % trimethylamine (Sigma-Aldrich). The permeation tubes were calibrated gravimetrically and the permeation oven was allowed to stabilize overnight prior to the measurements. The concentration levels for the undiluted samples were in the low ppm_v_ range (Table 1). This was chosen in order to achieve a concentration range that resembled those found in animal houses as much as possible, but at the same time to take the detection limits of the PTR-MS instrument at low detection dwell times into consideration. Hence, it should be noted that these compounds would be present at lower concentration levels in real samples.

### 2.2. Measuring Method

**Figure 1:**
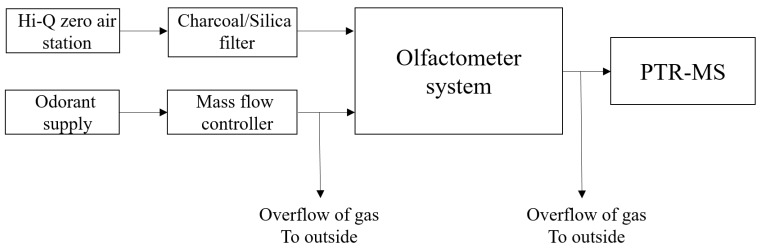
Schematic diagram of the principal experimental setup.

Figure 1 shows a schematic diagram of the principal setup used in all experiments. Dilution air for all the experiments and olfactometers was supplied through a charcoal/silica gel filter to provide clean and dry air. The air for the permeation oven and for the dilution air to bring the odorants to the desired concentrations was supplied from a HiQ zero air station (Linde AG, Munich, Germany). Air and odorant flows were supplied through Teflon tubes and were controlled by mass flow controllers (Bronkhorst, Ruurlo, The Netherlands), which were flushed with sample air for at least 1 h prior to the measurements. Samples were introduced to the olfactometer inlet with descending dilution factors and a blank sample between each to ensure stable background measurements. The expected concentration from which the recovery was estimated was defined as the inlet concentration measured by PTR-MS divided by the dilution factor. All dilution levels were repeated three times and the results are presented as average values of these triplicate determinations.

### 2.3. Olfactometers and Material Tubes

The olfactometer dilution systems were acquired from different accredited odor laboratories in Europe (Denmark, Germany and France); all had previously been used in commercial odor measurements and were made in accordance with the European standard for dynamic olfactometry [1]. 

#### 2.3.1. Glass Olfactometer

The glass olfactometer was made entirely of glass tubes with an inner diameter of 428 mm; it was approximately 1.5 m in length, and had four sniffing ports. The olfactometer had a sample introduction system consisting of Teflon tubes connected to a sample bag and a stainless steel needle. This system, however, was bypassed in the measurements for which the sample air was introduced directly from the permeation oven or standard gas cylinder connected with a small piece of flushed PTFE tubing. Hence, in these experiments, all parts of the olfactometer in contact with the sample during dilution were made of glass. The flow in the dilution system was 60 L min^−1^, equivalent to a flow of 15 L min^−1^ for each nose mask. The dilution factors included were 4480 to 140 times dilution and the pulse duration was set to 2 min. The normal pulse duration applied for panelist assessments with this olfactometer is 15 s. The olfactometer was tested on-site at the odor laboratory.

#### 2.3.2. Olfacton Dilution System

The Olfacton dilution system consisted of a 2.0 L min^−1^ mass flow controller (Bronkhorst, Ruurlo, The Netherlands) and connecting PTFE tubes coupled in a small portable unit. The parts in contact with the sample during dilution were the PTFE (tubing) and stainless steel (mass flow controller and fittings). This unit was normally used as a pre-dilution step to a larger distribution system with nose masks for panelists. In these measurements, however, only the dilution unit was tested. Dilution factors included were 3574 to 125 times dilution and the pulse duration tested was 60 s.

#### 2.3.3. TO8 Olfactometer

The TO8 instrument was provided and manufactured by Olfasense GmbH (Kiel, Germany). The dilution system was constructed of gas jet pumps and orifices for the sample dosage. The parts of the olfactometer in contact with the odorants were made of stainless steel (orifices) and PTFE (tubing). Dilution air for the olfactometer was provided by a compressor (Dr. Sonic, Fini, Bologna, Italy) and the flow in each nose cone was 20 L min^−1^. The olfactometer was calibrated with propane by Olfasense GmbH (Kiel, Germany) prior to the measurements. The dilution factors included were 3574 to 125 times dilution. The pulse durations tested were 15 and 60 s (cf. Section 2.4). A typical pulse duration applied for panelists during odor analysis is 2.2 s.

### 2.4. Material Testing

To clarify in which part of the TO8 losses occurred, the concentrations of odorants were determined at two points. In cooperation with the manufacturer (Olfasense GmbH, Kiel, Germany), a measuring point was added after the dilution system. The dilution system consisted mainly of orifices of stainless steel. For each odorant, the concentration at each dilution step was determined at this point with 15 s pulses. Afterwards, this point was closed tightly and the concentration of odorants at each dilution step throughout the full system was determined at the outlet to the nose cone using 60 s pulses. This allowed for a comparison of the loss taking place in the dilution system, which consisted mainly of stainless steel, and the distribution system, which consisted mainly of PTFE tubing.

To further test the influence of the olfactometer material, four tubes of PTFE, perfluoroalkoxy (PFA), stainless steel and SilcoTek-coated material were acquired. Each tube was 1.5 m in length with an inner diameter of 8 mm. The odorants were introduced through a dilution system consisting of PTFE tubes, mass flow controllers (Bronkhorst, Ruurlo, The Netherlands) and stainless steel fittings. The dilution system was allowed to equilibrate until the measured concentration of the odorants was equivalent to the expected value based on the undiluted concentration of the odorant, and the outlet of the system was subsequently connected to each of the tubes with descending dilution levels. The recovery of odorants through each material was measured directly at the outlet of each tube by PTR-MS. The flow of the sample and dilution air was kept at 20 L min^−1^.

### 2.5. PTR-MS

All measurements of the olfactometer output were obtained by a high-sensitivity PTR-MS instrument (Ionicon Analytic, Innsbruck, Austria). In this study, an ion drift tube voltage of 600 V was applied, the pressure was maintained at close to 2.2 mbar, and the temperature was set to 90 °C. This corresponded to a E/N ratio of 147 Td (E/N: electrical field per molecule number density). A ~1 m polyether ketone (PEEK) tube with dimensions of 1.6 mm OD and 0.64 mm ID and a flow of 250 mL min^−1^ was used for the inlet of the PTR-MS system. Dwell times were set to 200 ms for the primary ion and the odorants. For trimethylamine, the dwell time was increased to 500 ms because of a poorer response for this compound. This resulted in cycle times of 400 and 700 ms, respectively. The PTR-MS inlet was placed in the middle of the outlet to the sniffing ports of the TO8 and glass olfactometers, while for the testing of the materials and tubes, the inlet was connected with a short 8 mm PTFE tube coupled with an overflow tube to remove excess gas.

Table 1 shows the assigned *m*/*z* values for each odorant and their detection limit, defined as three times the standard deviation of the noise signal. Carboxylic acids will typically fragment by losing one water molecule [16]. Hence, the concentrations of carboxylic acids were estimated as the sum of the protonated-parent *m*/*z* signals at 61, 89, 75, and 103, for acetic acid, butanoic acid, propanoic acid and 3-methylbutanoic acid, respectively, and their fragments at 43, 71, 57 and 85, respectively.

### 2.6. Data Analysis

For hydrogen sulfide, the correction method described by Feilberg et al. [16] was applied. This was necessary because the proton transfer reaction that takes place in the drift tube of the PTR-MS is only energetically possible for VOCs with a proton affinity greater than that of water (691 kJ mol^−1^). Because hydrogen sulfide only slightly exceeds this value (705 kJ mol^−1^), a humidity-dependent backward reaction of protonated hydrogen sulfide becomes significant. A power function was found as the best fit for the conditions applied in this study (R^2^ > 0.9822).

For the carboxylic acid, 4-methylphenol, and trimethylamine in particular, a delay in the signal rise and fall-off time was observed when these were measured directly with the PTR-MS system. A delay of adsorptive compounds in the PTR-MS system has previously been reported [19,20,21,22]. To eliminate the effect on the odorant breakthrough curves, which was caused by the PTR-MS system, all compounds were measured directly with quadruple determination by PTR-MS. For these measurements, an automatic three-way valve, which was connected to—and controlled by—the PTR-MS instrument, was added for immediate compound dosage to estimate the true response time of the PTR-MS signal. For each compound, five different dilution steps were included, and the compound concentrations were kept at the same level as in the olfactometer experiments. 

The data obtained for the signal response and tail-off could be modelled with an adequate fit by means of a non-linear least squares regression with a sigmoid curve and an exponential decay function of the type given in Equations (1) and (2), respectively:

(1) $$C_{s\;\left(\mathrm{response}\right)}=C_{\exp}\left(1-e^{-\mathrm{tB}}\right)$$

(2) $$C_{s\;\left(\mathrm{tail}\right)}=C_{\exp}\cdot e^{-\mathrm{tB}}$$

where C_s_ is the concentration signal measured by PTR-MS, C_exp_ is the inlet odorant concentration, t is the time and B is a fitted parameter characterizing the slope of the curve and, thus, the time before the signal plateau at C_exp_ or the background level is reached. Table 1 lists the B-parameters fitted for the data and the average model fit.

In the following analysis, these models were used to estimate the expected PTR-MS signal equivalent to a certain time and inlet concentration.

## 3. Results and Discussion

**Figure 2:**
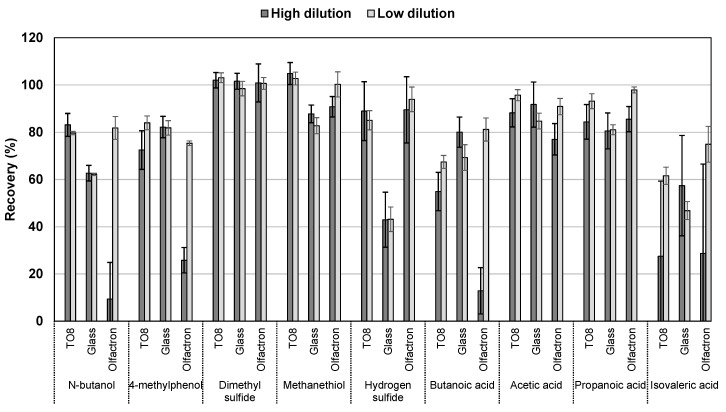
Maximum recovery for a high (dilution factor of 1300) and a low (dilution factor of 250) dilution in the TO8 olfactometer, the glass olfactometer and the Olfacton dilution unit at the end of a 15 s pulse.

Figure 2 displays the maximum recovery for a high (1300 times) and a low (250 times) dilution in the three different olfactometers at the end of a 15 s pulse (estimated by the average of the last five cycles, equivalent to ~2.8 s). In the PTR-MS system, all compounds except trimethylamine had a recovery of above 95% after 15 s. Hence, losses exceeding 5% may have been attributed mainly to the olfactometer. It is evident from Figure 2 that the olfactometer played a significant role in the recovery of odorous compounds, even at the longest pulse of 15 s as recommended by the European standard. For the compounds tested, the recovery was below 90% of the expected inlet value in 79.3% and 65.5% of the cases for the highest and lowest dilution, respectively. For the highest dilution, 27.6% were below 60%. Considering these results, it must be stressed that the highest dilutions were the most representative for real odor samples found in animal production, and the most adsorptive compound, trimethylamine, was not included in these calculations (cf. Section 3.4).

Considering all the dilution factors and compounds, the TO8 olfactometer had a slightly higher overall recovery (81.9%) than the glass olfactometer (74.2%), which was statistically significant (*p* = 0.026). The overall performance of the Olfacton dilution system was similar to the glass olfactometer, and had an average recovery of 74.2%, but the variability of this system was considerably higher, spanning from an average recovery (including all compounds) of 57.8% to 88.5% for the highest to the lowest dilution, respectively. Figure 2 clearly shows that not only the overall recovery and variability between the dilution levels differed between the three systems, but there was also a significant difference in the behavior of different compounds in relation to the olfactometer system.

### 3.1. n-Butanol

**Figure 3:**
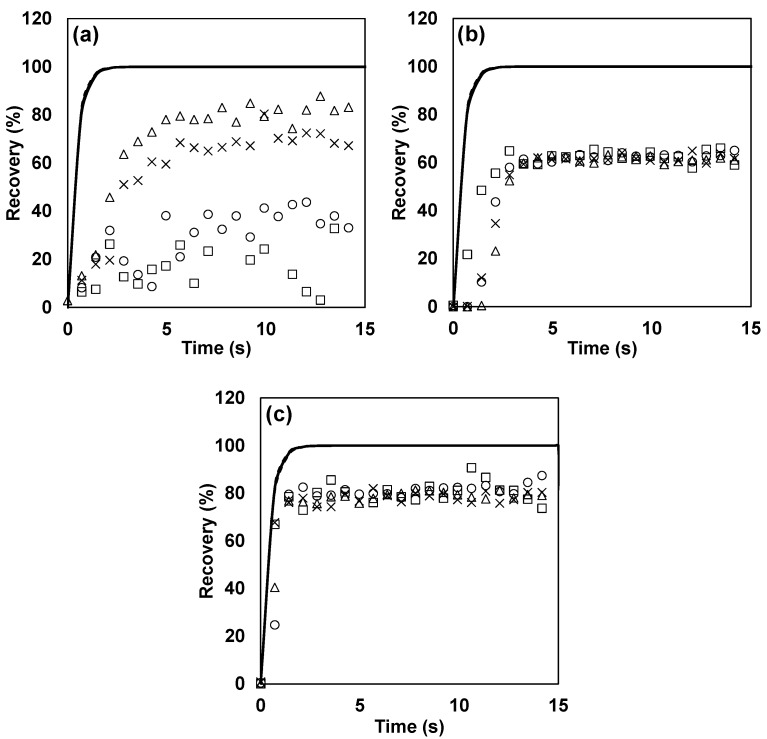
Evolution of recovery of *n*-butanol in: (**a**) Olfacton dilution unit, (**b**) glass olfactometer, and (**c**) TO8 olfactometer. The solid line (—) indicates the expected recovery on the basis of the fitted PTR-MS model, the dotted line (······) indicates the 95% confidence interval of the model, □ represents a dilution factor of ~1600, ○ represents a dilution factor of ~800, × represents a dilution factor of ~400, and ∆ represents a dilution factor of ~200.

A notable result from this study was the difference in the recovery of *n*-butanol in different systems. In the TO8 olfactometer, on average, 79.9% of the initial inlet value was recovered, while only 63.1% and 49.9% were recovered in the glass olfactometer and in the Olfacton dilution system, respectively. Furthermore, a strong dependence on the dilution level was observed in the Olfacton unit, for which the recovery for the highest dilution was only 9.4%, which steadily increased to 81.8% for the lowest dilution. This strong dependence on the dilution factor suggested a high level of adsorption or surface reactivity and, thus, saturation effects taking place. This is supported by the fact that *n*-butanol was found to adsorb strongly to stainless steel (cf. Section 3.7). According to the European standard for dynamic olfactometry, *n*-butanol is the compound used to select panelists. Hence, varying degrees of recovery of this compound in different types of olfactometers may influence the outcome and reproducibility of odor measurements performed at different laboratories with different types of olfactometers, even if these are built and operated within the limits of the European standard for dynamic olfactometry. Figure 3 shows the evolution of recovery during the first 15 s for *n*-butanol in the Olfacton, the glass olfactometer and the TO8 olfactometer. It is evident that the recovery in the Olfacton was impaired for the highest dilutions and that the signal plateau varied between the different systems.

The fact that the recovery of *n*-butanol stabilized relatively quickly at a plateau below the expected value and that the mass lost was not recovered by tailing (i.e., the integrated area below the curve of the measured concentration did not match or approach that of the expected concentration in any cases) indicated that the loss was partly due to a reactive removal process; however, in the glass olfactometers, this may also have been caused by flow conditions and the position of the measuring point (cf. Section 3.7). It has not been possible to recreate the reactive loss in glass in subsequent experiments in glass impingers, although significant adsorption effects were also observed (data not shown). However, these effects may have been a result of the specific surface treatment of the glass and possibly fouling layers from previously run samples. Hence, more research is needed to clarify this issue.

### 3.2. Sulfur Compounds

The reduced sulfur compounds had the highest recovery in all cases, reaching 96.4%, 76.4%, and 98.1% in the TO8 olfactometer, the glass olfactometer, and the Olfacton dilution system, respectively. However, dimethyl sulfide was the only compound that was almost completely recovered in all cases. Methanethiol and hydrogen sulfide were subject to the relatively significant losses in the glass olfactometer of approximately 20% and 60%, respectively. This was consistent with previous studies, which found that sulfur compounds (i.e., hydrogen sulfide) undergo losses at the surface of sample materials of glass and stainless steel [23,24,25]. The loss found in this study followed the pattern of reactivity of hydrogen sulfide being the most reactive of the tested sulfur compounds and dimethyl sulfide being a non-protic and relatively non-reactive compound. In relation to hydrogen sulfide, it should be noted that the sensitivity of the measurements was reduced because of the low raw signal of this compound before a humidity correction. This resulted in a relatively higher standard deviation at the plateau of, on average, ±10−15% compared to the other sulfur compounds, which alongside *n*-butanol, showed a high level of stability in the PTR-MS measurements by the high B-values, little to no tail-off, and stable signals at the concentration maxima (±2−3%).

**Figure 4:**
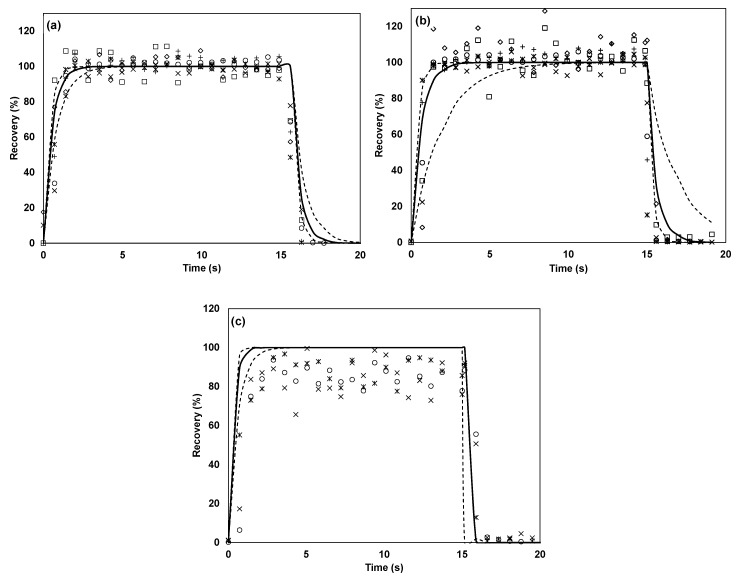
Recovery in TO8 olfactometer of: (**a**) dimethyl sulfide, (**b**) methanethiol, and (**c**) hydrogen sulfide. The solid line (—) indicates the expected recovery on the basis of the fitted PTR-MS model, the dotted line (······) indicates the 95% confidence interval of the model, □ represents a dilution factor of 3574, ◇ represents a dilution factor of 1958, + represents a dilution factor of 970, × represents a dilution factor of 483, ꁘ represents a dilution factor of 236, and ○ represents a dilution factor of 125.

Figure 4 depicts the recovery of the reduced sulfur compounds in the dilution system of the TO8 olfactometer with 15 s pulses and the expected recovery based on the B-parameter fitted to the PTR-MS instrument response. The same trends in the evolution of the recovery were found in the other systems, and only the level at which the plateau stabilized differed between the systems. As for *n*-butanol, the level of tailing could not account for the mass lost, indicating a reactive loss for hydrogen sulfide, in particular in the glass olfactometer, where methanethiol was also not fully recovered. Hydrogen sulfide and methanethiol displayed some degree of adsorptive behavior in the TO8 olfactometer and the Olfacton dilution system. This, however, was limited compared to the other compounds tested, and because of the relatively fast breakthrough of the sulfur compounds, the pulse duration presumably had little influence on the odor measurements. The sulfur compounds tested reached >90% of their maximum within a 15 s pulse and >80% even within the short pulse duration of 2.2 s, which was typically implemented with the TO8 olfactometer.

### 3.3. Carboxylic Acids and 4-Methylphenol

Considering carboxylic acids, the performances of the olfactometers were comparable; there was no statistically significant difference between them. The average recovery (considering all dilutions) was 72.1%, 73.8% and 68.7% for the TO8 olfactometer, the glass olfactometer and the Olfacton dilution system, respectively. For 4-methylphenol, the performance of the TO8 olfactometer and the glass olfactometer was also almost equal (~80%), while the Olfacton dilution system showed a reduced average recovery of 48.6% and significant saturation effects. 

**Figure 5:**
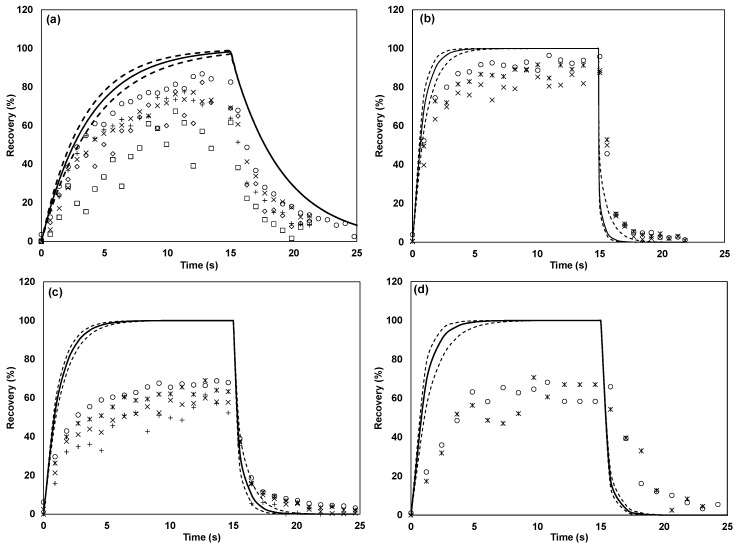
Recovery in TO8 olfactometer of: (**a**) 4-methylphenol, (**b**) propanoic acid, (**c**) butanoic acid, and (**d**) 3-methylbutanoic acid. The solid line (—) indicates the expected recovery on the basis of the fitted PTR-MS model, the dotted line (······) indicates the 95% confidence interval of the model, □ represents a dilution factor of 3574, ◇ represents a dilution factor of 1958, + represents a dilution factor of 970, × represents a dilution factor of 483, ꁘ represents a dilution factor of 236, and ○ represents a dilution factor of 125.

Carboxylic acids, 4-methylphenol, and trimethylamine had relatively slow initial responses (i.e., low B-values) and showed varying degrees of tailing. For most dilution levels, these compounds did not reach a stable plateau within 15 s, which is the maximum pulse duration of the European standard. Figure 5 shows the recovery of 4-methylphenol, butanoic acid, propanoic acid and 3-methylbutanoic acid with different dilution levels in the TO8 dilution system. Acetic acid and propanoic acid were found to behave similarly (only propanoic acid is shown). For all the compounds, similar trends were observed in the other olfactometer systems.

Because of the slower response and time-dependent reduction of the recovery of carboxylic acids and 4-methylphenol, the pulse duration had a significant influence on the outcome of the measurements. Table 2 shows the recovery after 2.2, 15 and 60 s pulses with a dilution factor of ~400. The pulses at 15 and 60 s were estimated by the last five cycles, while the recovery at 2.2 s was estimated as the average of the cycles falling between 1.8 and 3 s (~3 cycles). The total average recovery is shown at the end of Table 2. It is evident that a longer pulse duration provides results that are more accurate, and a short pulse, as for at 2.2 s, may lead to an underestimation of the odor concentration, especially when considering the fact that odorants are found in significantly lower concentrations in real odor samples; it thus may be expected that these effects will be augmented (cf. Section 3.6).

### 3.4. Trimethylamine

Trimethylamine demonstrated particularly adsorptive characteristics, making the measuring and data treatment difficult. In the preliminary performance assessment of odorants in the PTR-MS system, trimethylamine was fitted with the lowest B-value of 0.11, meaning that this compound reached 90% of the inlet concentration after 20 s. Because of this and because of the low concentration range applied in this study, it was not possible to measure this compound confidently. In the glass olfactometer, this compound was not only significantly delayed in the response, but it was also possible to measure traces of it several hours after the addition to the system (Figure S1 in the Supplementary Materials). In the TO8 olfactometer and Olfacton system, this compound was below the detection limit or the signal was delayed to an extent for which it was not possible to differentiate properly between the different dilutions. This shows that great care should be taken when measuring odor samples containing amines and other highly adsorptive compounds. 

### 3.5. Factors Influencing Adsorption and Recovery

**Figure 6:**
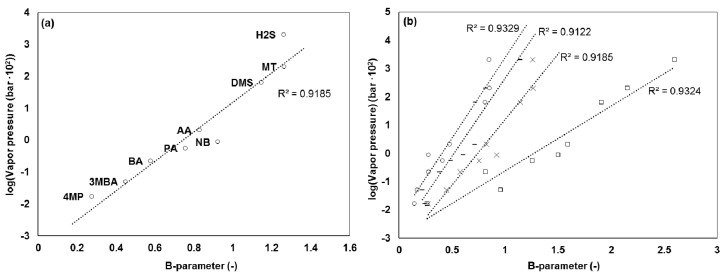
(**a**) B-values of compounds in TO8 dilution system versus the logarithm of their vapor pressure (4MP = 4-methylphenol, 3MBA = 3-methylbutanoic acid, BA = butanoic acid, PA = propanoic acid, AA = acetic acid, NB = *n*-butanol, DMS = dimethyl sulfide, MT = methanethiol, and H2S = hydrogen sulfide), (**b**) B-values of compounds in PTR-MS (□), TO8 olfactometer (×), glass olfactometer (︻), and Olfacton dilution system (○). Data labels are omitted for simplicity but follow the order of Figure 6a.

On a general basis, the main factors determining the level of physical adsorption are the temperature, the gas vapor pressure and the available surface area. Hence, because the temperature and available surface area were constant in each system, some correlation to the vapor pressure was expected. As for in previous studies [7], no correlation between the recovery and vapor pressure could be made directly (0.02 < R^2^ < 0.6). However, the recovery may have been compromised by several different factors other than the physical adsorption, for example, reactive losses due to chemical interactions in the system, which are a function of specific chemical properties in relation to the composition of the adsorptive material and sample air matrix. Furthermore, a relatively low sensitivity for certain compounds, such as 3-methylbutanoic acid, may have affected the precision of the measurement, especially for very low inlet concentrations, increasing the uncertainty of the true recovery. Instead, to obtain a measure of the adsorption in the system, a partitioning coefficient could be defined as the difference in the recovery per unit time, that is, the slope of the curve after the compound breakthrough or the tail, which in this case was estimated by the B-parameter, as determined earlier. This parameter defines the amount of a compound delayed by the system as a result of adsorption. Figure 6a shows the relation between the B-parameter and the vapor pressure in the TO8 dilution system (R^2^ = 0.92). In all cases (i.e., for the glass olfactometer (R^2^ = 0.91) and the Olfacton (R^2^ = 0.93)) there was a similar relation. Figure 6b shows that the magnitude of the B-values decreased in the order: TO8 olfactometer, glass olfactometer, Olfacton dilution system.

### 3.6. Saturation Effects

**Figure 7:**
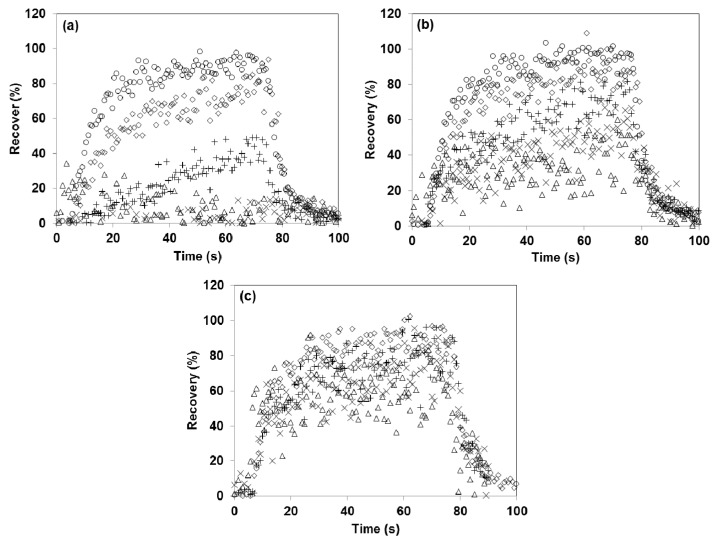
Saturation effects on 4-methylphenol concentration during replicates of dilution series in Olfacton system. (**a**) First repetition, (**b**) second repetition, and (**c**) third repetition; ∆ represents dilution factor of 1995, × represents dilution factor of 1313, + represents dilution factor of 751, ◇ represents dilution factor of 365, ○ and represents dilution factor of 208.

Normally, during dynamic olfactometry, the samples are presented in an ascending order, whereby samples with the highest dilution are presented before samples with a lower dilution. In this study, it was found that the concentration at high dilutions was significantly lower than expected, especially for the first samples run on a clean instrument. As an example, Figure 7 shows three replicates of a dilution series with 4-methylphenol in the Olfacton dilution system. The three replicates were performed sequentially and uninterruptedly with a blank sample between each sample and between each of the three dilution rows. It is apparent that adsorption to the inner surfaces caused the first low concentration samples to show significant losses compared to the expected values, while, as the surfaces became saturated with this compound, this effect decreased, and the recovery of all diluted samples approached the expected value in the last replicate. Similar saturation effects were observed to varying degrees for all carboxylic acids, 4-methylphenol, *n*-butanol and methanethiol in the Olfacton dilution system, and to a lesser extent in the TO8 olfactometer. In the glass olfactometer, this trend was not observed to a significant extent, presumably because of the much smaller surface area-to-sample air volume ratio. 

It is noteworthy that saturation effects also influenced the results of this study. All the recoveries were presented as the averages of three replicates and, thus, as seen from Figure 7, we may likely have overestimated the actual recovery found in the odor analysis. The first replicate is the most representative of the real situation in odor laboratories, as odorants in a low concentration are presented first when the instrument is clean. Furthermore, it is also necessary to view the results of this study in relation to the inlet concentrations of the compounds (Table 1). As *n*-butanol, for example, was introduced into the olfactometers in a higher initial concentration (20 ppm_v_), the tendency of this compound to stick to the olfactometer may have been underestimated when comparing it to that of the other compounds introduced at lower concentrations. In this case, the higher inlet concentration caused an equilibrium in the system to set in earlier. This resulted in a higher B-value compared to other compounds introduced at lower concentrations, as also observed in Figure 6a. The concentration of 20 ppm_v_ was, however, representative of that which is used during odor evaluations.

One way to overcome the issue of adsorption and saturation effects could be to flush the system with the sample air prior to the measurements and/or increase the pulse duration considerably so that an equilibrium state with the specific concentration of the sample may set before the measurements are performed. However, this may not be a viable solution in the case of environmental samples, for which the concentration levels are in the low ppb range, as this may necessitate excessively large samples.

### 3.7. Effect of Materials and Composition

**Figure 8:**
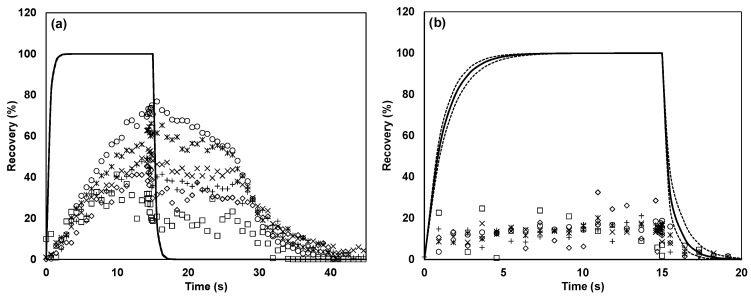
Recovery of (**a**) *n*-butanol and (**b**) butanoic acid in stainless steel tube. The solid line (—) indicates the expected recovery on the basis of the PTR-MS fitted model, the dotted line (······) indicates the 95% confidence interval of the model, □ represents a dilution factor of 3574, ◇ represents a dilution factor of 1958, + represents a dilution factor of 970, × represents a dilution factor of 483, ꁘ represents a dilution factor of 236, and ○ represents a dilution factor of 125.

In the TO8 olfactometer, the recovery in the complete system was found to be approximately equal to the recovery in the dilution system or even larger (presumably because of saturation effects, as the dilution system was tested first). This suggests that the main loss took place in the dilution system, which consisted mainly of stainless steel. Figure 8 depicts the recovery of *n*-butanol and butanoic acid through a stainless steel tube, and it is apparent that the recovery was significantly affected in both cases. 

Similar effects, although to varying degrees, were observed for all carboxylic acids and 4-methylphenol, while hydrogen sulfide and methanethiol showed minor losses, and dimethyl sulfide was unaffected. No significant loses were observed for PTFE, PFA and SilcoTek-coated steel (Figures S2 and S3 in the Supplementary Materials). 

**Figure 9:**
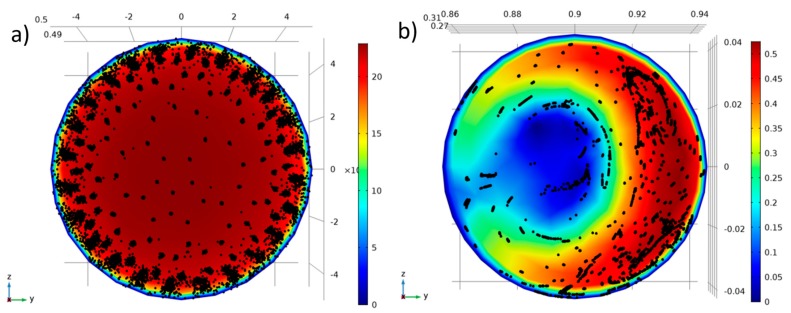
Particle trajectories predicted by numerical solution of flow conditions in COMSOL Multiphysics at the outlet of: (**a**) the TO8 olfactometer and (**b**) the glass olfactometer. Dimensional units are in meters and the velocity scale bars are in m/s. Dimensional units are in millimeters in (**a**) and in meters in (**b**). The velocity color scale bars are in meters per second.

The design of the olfactometers may also play a role in the recovery of odorants. In this study, the olfactometers examined varied greatly in their dimensions. Comparing the delivery lines in the glass olfactometer to those applied in the TO8 olfactometer and the Olfacton dilution system, the cross-sectional area and the specific surface area were approximately 28 and 5 times greater, respectively, in the glass olfactometer. Furthermore, the glass olfactometer was constructed with two 90° bends. The possible influence of flow and transport phenomena on the delivery of odorants to the outlet of the TO8 and glass olfactometers were investigated numerically with Comsol Multiphysics. The Navier–Stokes equations describing the fluid dynamic problem arising when 80 L/min of air enters each of the pipe systems and exits at atmospheric pressure were solved as a stationary problem using the direct Paradiso algorithm. The flow was turbulent with Reynolds numbers of approximately 3000 for the glass olfactometer and 57.000 for the TO8 olfactometer. Therefore, a RANS Spalart–Allmaras model was used to describe the turbulent flow behaviour. Supplemental Figure S4 (in the Supplementary Materials) shows the velocity field at a section through the middle of the glass olfactometer, and stream lines describe the 3D flow through the system. To numerically mimic the overall interaction between the convective flow and individual molecules, massless particles were released at the inlet and tracked through the delivery lines. The adsorptive behavior of the particles was not considered in this model. The result in Figure 9 is a spatial plot of the expected distribution of the molecules, together with the absolute velocity of the flow at the exit.

As seen from Figure 9, the numerical solution suggests that the spatial distribution of odorant particles at the outlet in the glass olfactometer was impaired because of recirculation zones imposed by the bends (Figure S4 in the Supplementary Materials). On the other hand, the model predicted that this would not have been an issue in the TO8 olfactometer. The spatial distribution of the concentration and the air velocity may influence the gas-wall contact and transfer velocity. To clarify the exact impact of these phenomena on odor measurements, more research is needed. Furthermore, the effect of the nose cone needs to be taken into consideration [7]. However, these preliminary results strongly suggest that the impact of the configuration of the olfactometer should be examined further.

## 4. Conclusions

On the basis of the results of the present study, it is found that three different olfactometers obtained from three accredited odor laboratories produced different results in relation to odorant recovery. Significant losses and saturation effects were recorded for high dilutions, particularly in stainless steel. Furthermore, the results show that not only the overall recovery and variability between dilution levels differed between the three olfactometer systems; there was also a significant difference in the behavior of different compounds in relation to the olfactometer system. This makes the prediction of the behavior of real odor samples, in which the chemical composition is complex—and in many cases, unknown—difficult. A notable result from the study is the adsorptive behavior of *n*-butanol, which is the reference compound appointed by the European standard for dynamic olfactometry, EN13725. Varying degrees of recovery of this compound in different types of olfactometers may influence the outcome and reproducibility of odor measurements performed at different laboratories with different types of olfactometers, even if these are built and operated within the confinements of the European standard. Hence, to maintain the credibility and improve the reproducibility of dynamic olfactometry in the evaluation of environmental samples, it is the conclusion of the present study that further and stricter standardization is necessary. Stainless steel and glass should be omitted from sampling equipment exposed to odorants similar to those tested, while the use of PTFE, PFA or SilcoTek-coated steel is evaluated as a better choice. In addition, the configuration of the olfactometers should be considered.

## Figures and Tables

**Table 1:** Assigned *m*/*z* values, fitted B-parameter, inlet concentrations, detection limits and average model fit.

Compound	*m*/*z* Value	Detection Limit (ppb)	Inlet Concentration (ppm_v_)	Fitted B-Value ± 95% Confidence Interval	Average Model Fit (R^2^)
Trimethylamine	60	0.55	2.7 ± 0.1	0.11 ± 0.018	0.94
4-Methylphenol	109	0.50	3.6 ± 0.2	0.27 ± 0.16	0.82
Butanoic acid	89 + 71	0.41	3.2 ± 0.2	0.82 ± 0.14	0.81
3-Methylbutanoic acid	103 + 85	0.29	5.3 ± 0.7	0.96 ± 0.29	0.70
Propanoic acid	75 + 57	0.20	3.2 ± 0.4	1.25 ± 0.31	0.90
Acetic acid	61 + 43	1.0	4.4 ± 0.1	1.59 ± 0.36	0.98
Hydrogen sulfide	35	2.6	5.55 ± 0.55	2.60 ± 1.4	0.96
Methanethiol	49	0.06	5.15 ± 0.51	2.15 ± 1.1	0.98
Dimethyl sulfide	63	0.19	6.03 ± 0.30	1.91 ± 0.68	0.84
*n*-Butanol	57	0.37	20 ± 1.0	2.41 ± 0.13	0.99

**Table 2:** Recovery (%) of odorants in TO8 olfactometer, glass olfactometer, and Olfacton dilution systems at pulse durations of 2.2, 15 and 60 s. Results have been corrected for adsorption behavior in PTR-MS. The relative standard deviation (%) is shown in parentheses.

	TO8 Olfactometer			Glass Olfactometer			Olfacton		
	2.2 s	15 s	60 s	2.2 s	15 s	60 s	2.2 s	15 s	60 s
*n*-Butanol	68.1 (12.7)	76.7 (2.1)	98.8 (3.1)	33.9 (63.2)	62.2 (3.5)	61.7 (1.7)	29.5 (63.1)	70.0 (3.9)	77.6 (3.8)
4-Methylphenol	68.1 (12.7)	76.7 (2.1)	98.8 (3.1)	80.3 (13.4)	84.7 (3.1)	82.1 (4.7)	48.0 (5.8)	63.5 (2.4)	82.0 (1.8)
Dimethyl sulfide	97.3 (2.2)	97.6 (1.1)	97.0 (0.7)	63.0 (44.3)	100.5 (5.0)	104.1 (4.5)	97.3 (7.5)	99.2 (4.1)	100.9 (5.2)
Methanethiol	98.3 (1.0)	99.2 (3.8)	95.5 (9.8)	77.5 (8.3)	82.8 (3.4)	84.7 (3.3)	74.8 (22.3)	102.0 (5.8)	101.3 (3.4)
Hydrogen sulfide	87.0 (2.7)	80.6 (8.9)	95.5 (17.8)	44.8 (3.3)	43.2 (5.2)	47.5 (4.9)	58.7 (25.9)	95.7 (11.1)	89.0 (5.0)
Butanoic acid	45.1 (1.4)	57.8 (1.2)	79.4 (9.3)	49.5 (14.0)	69.3 (5.5)	73.2 (3.1)	33.9 (18.6)	70.0 (2.7)	77.6 (2.9)
Acetic acid	62.9 (13.6)	86.5 (2.6)	95.3 (1.8)	38.0 (26.6)	85.1 (6.8)	86.6 (4.8)	63.6 (9.6)	87.9 (4.3)	90.6 (2.9)
Propanoic acid	73.8 (6.8)	82.6 (2.7)	94.0 (8.8)	51.5 (17.3)	80.0 (2.0)	88.1 (2.2)	62.2 (18.5)	92.3 (2.0)	98.0 (3.4)
3-Methylbutanoic acid	25.0 (7.2)	43.2 (18.5)	72.0 (22.7)	20.6 (8.3)	42.8 (6.1)	55.6 (9.1)	<DL <DL	40.7 (11.2)	81.8 (15.0)
Average Recovery	69.5	77.8	91.8	51.0	72.3	76.0	52.0	80.1	88.8

## Supplementary Materials

The following are available online at http://www.mdpi.com/1424-8220/17/8/1859/s1, Figure S1: Inlet and outlet concentration of trimethylamine in glass olfactometer; Figure S2: Recovery of odorants when flushed through the TO8 connected to tubes of different materials; Figure S3: Recovery of odorants in tubes of SilcoTek-coated steel, stainless steel, PTFE and PFA; Figure S4: COMSOL model—Velocity field in glass olfactometer.
