# Supplementary material for: Effects of Dilution Systems in Olfactometry on the Recovery of Typical Livestock Odorants Determined by PTR-MS
Source: Sensors (Basel). 2017 Aug 11;17(8):1859. doi: 10.3390/s17081859 (PMC5579735; doi:10.3390/s17081859)
Supplement: Supplementary file 1 [file sensors-17-01859-s001.pdf]

sensors

Supplementary Information

Effects of Dilution Systems in Olfactometry on the Recovery of Typical Livestock Odorants Determined by PTR–MSD

Pernille Lund Kasper [1], Dietmar Mannebeck [2], Arne Oxbøl [3], Jens Vinge Nygaard [1], Michael Jørgen Hansen [1] and Anders Feilberg [1,*]

1. Department of Engineering, Aarhus University, Hangøvej 2, DK-8200 Aarhus N, Denmark; peka@eng.au.dk (P.L.K.); jvn@eng.au.dk (J.V.N.); michaelj.hansen@eng.au.dk (M.J.H.)
2. Olfasense GmbH, Fraunhoferstraße 13, DE-24118 Kiel, Germany; dmannebeck@olfasense.com
3. FORCE Technology, Park Allé 345, DK-2605 Brøndby, Denmark; aox@force.dk

* Correspondence: af@eng.au.dk; Tel.: +45-3089-6099

Received: 26 July 2017; Accepted: 7 August 2017; Published: date

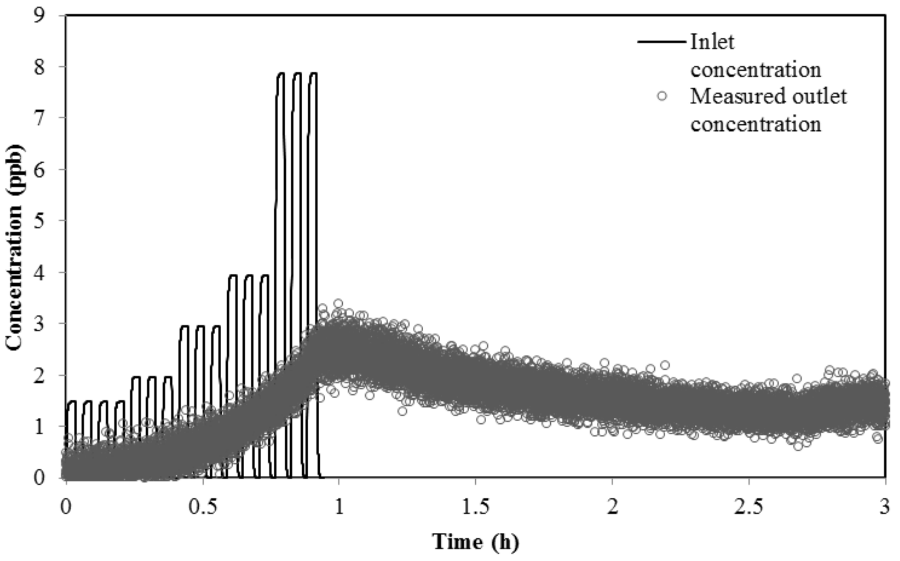

Supplemental Figure S1. Inlet and outlet concentration of trimethylamine in glass olfactometer

a)

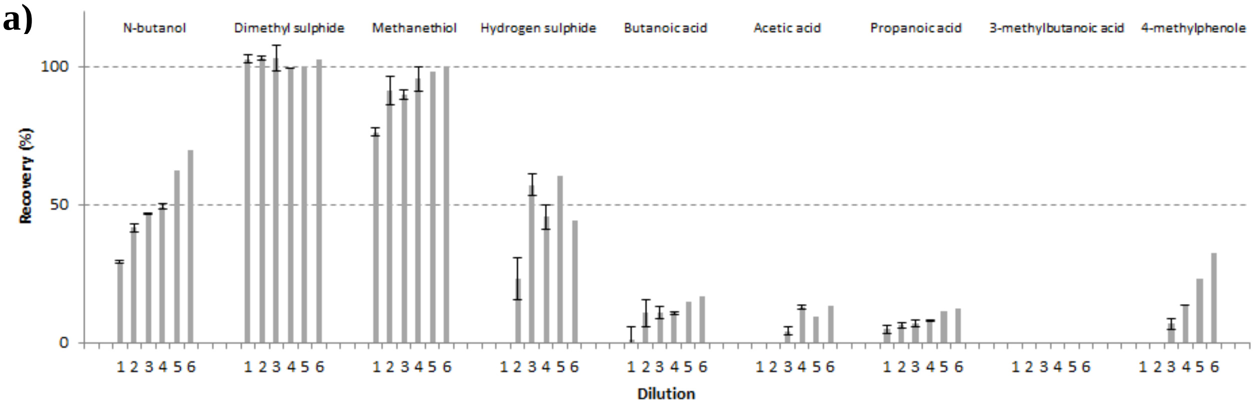

b)

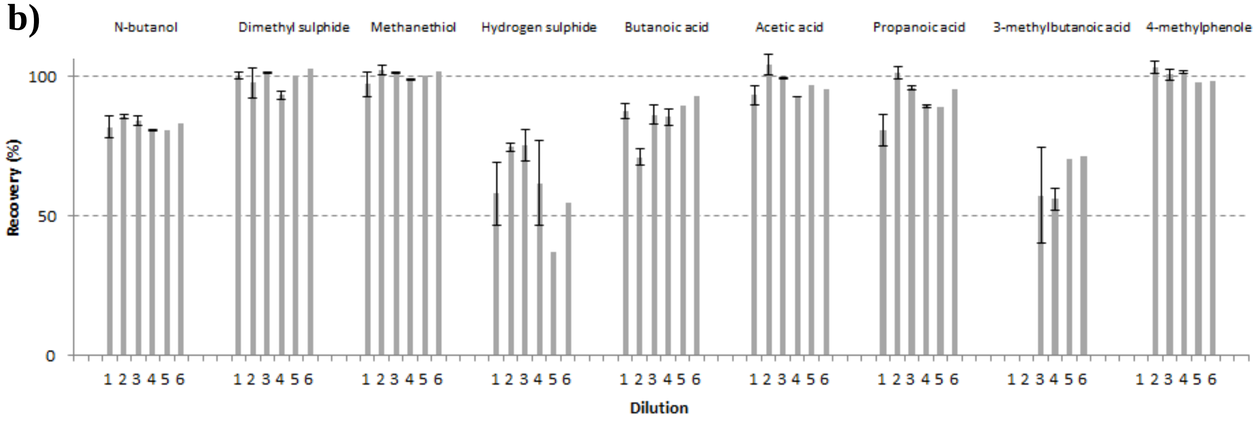

c)

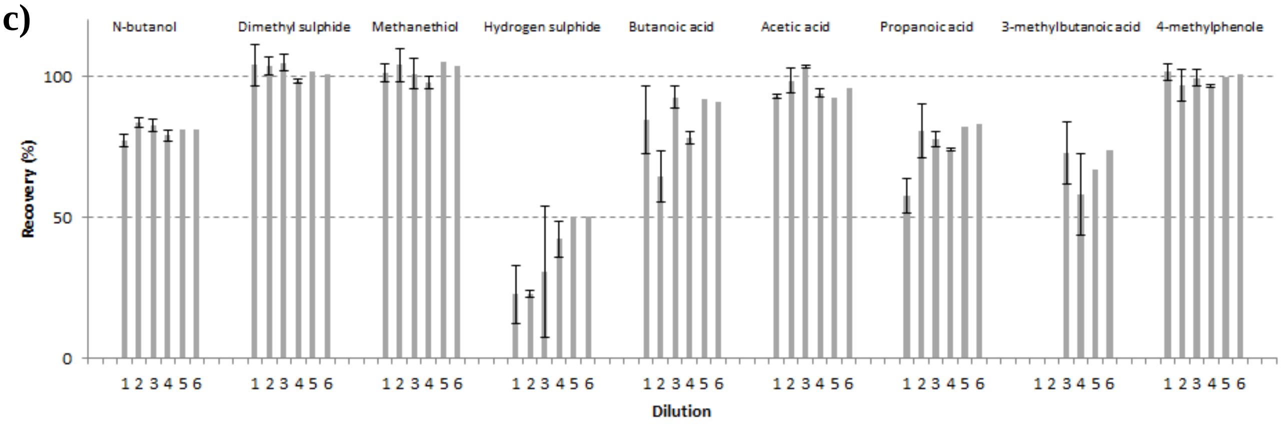

Supplemental Figure S2. Average recovery of odorants after 15 s. when flushed through the TO8 and a tube of a) stainless steel, b) PTFE, c) PFA. Numbers indicate dilution factor (1: 3571, 2:1958, 3:970, 4:483, 5:236, 6:125)

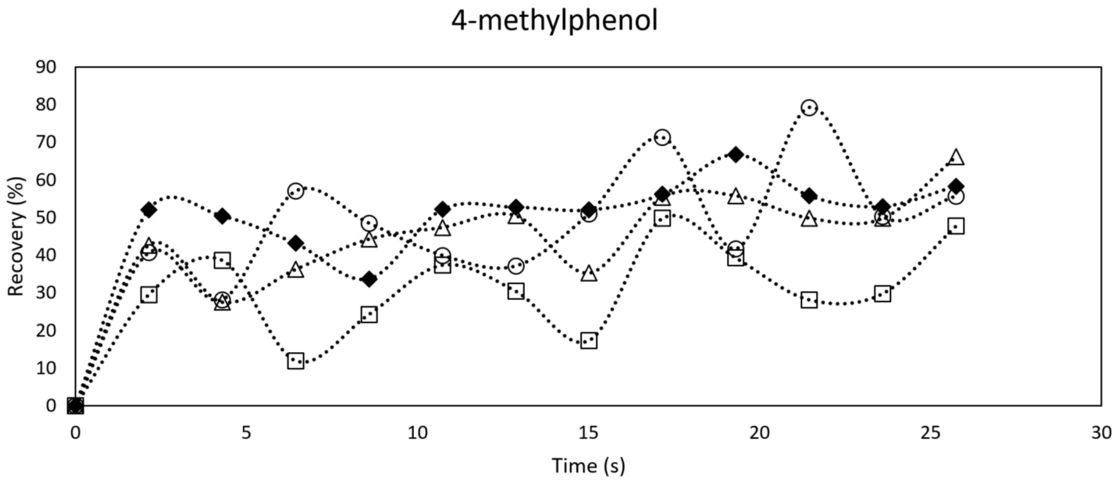

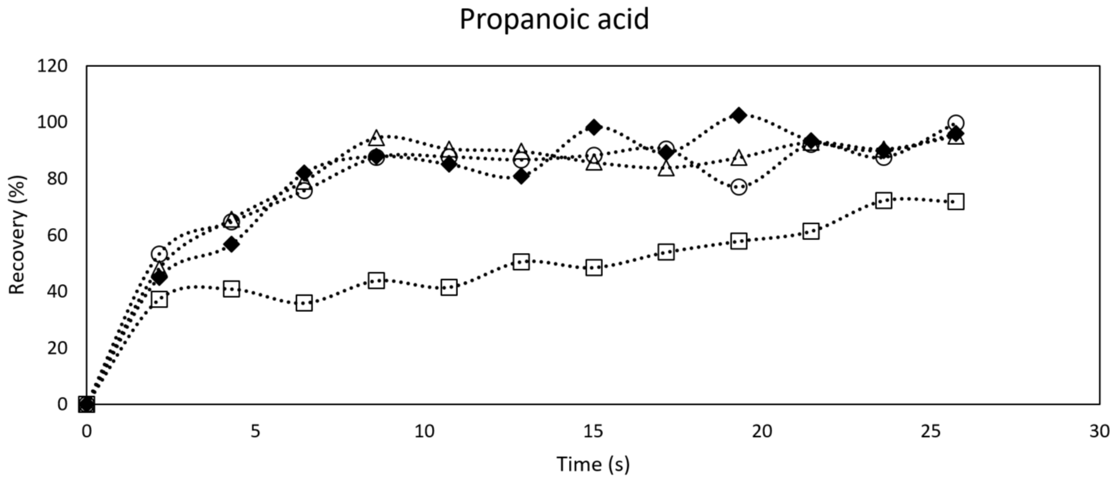
Propanoic acid
Recovery (%)
Time (s)
0
20
40
60
80
100
120
0
5
10
15
20
25
30

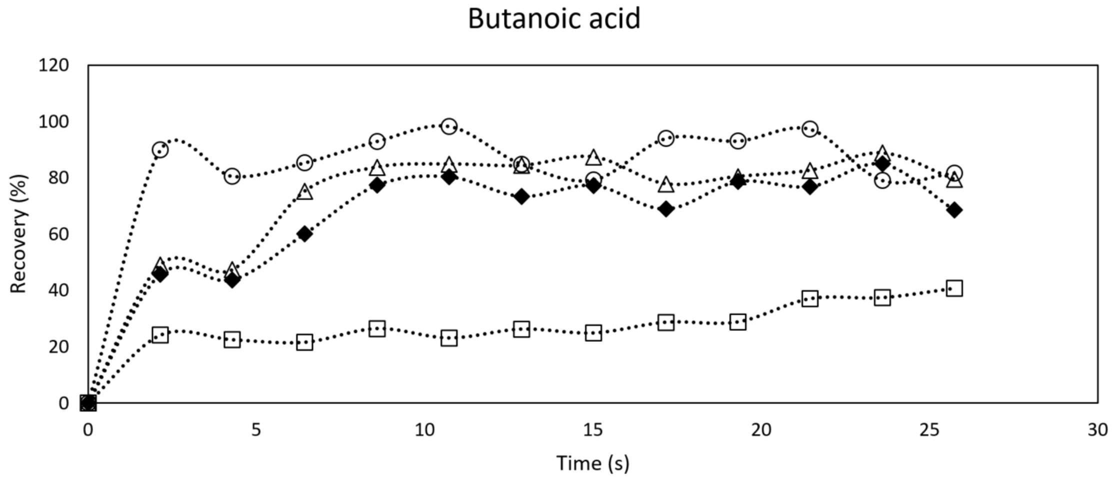
Butanoic acid
Recovery (%)
Time (s)
0
20
40
60
80
100
120
0
5
10
15
20
25
30

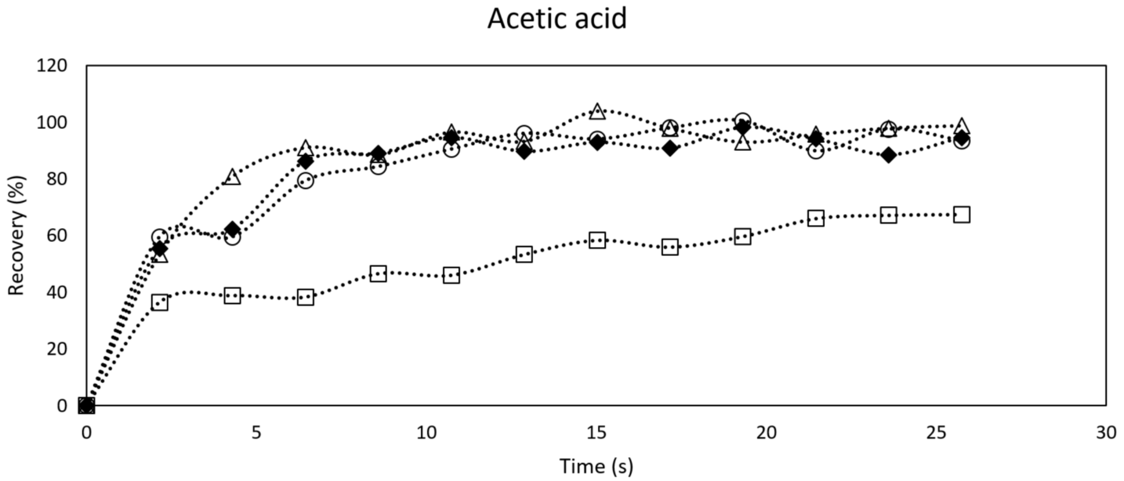
Acetic acid
Recovery (%)
Time (s)
0
20
40
60
80
100
120
0
5
10
15
20
25
30

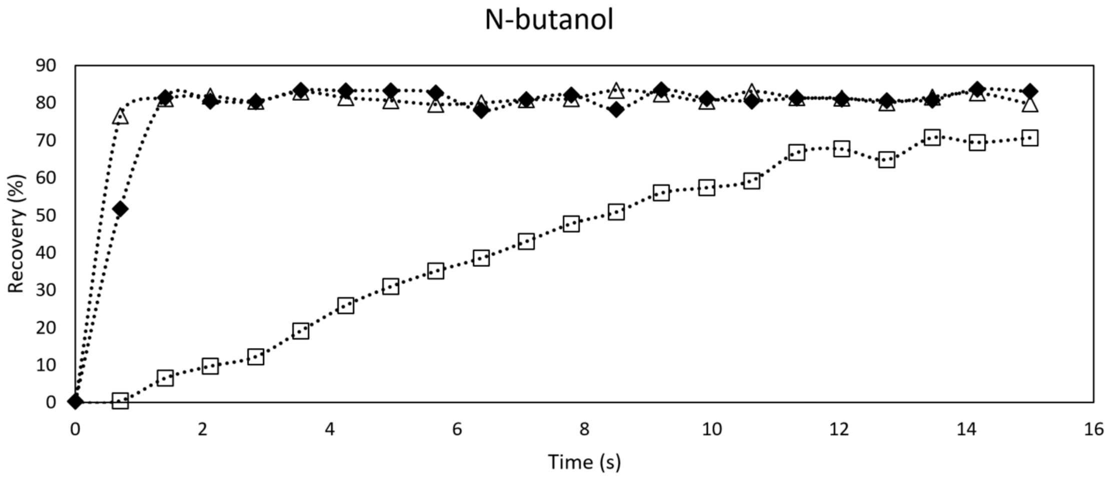
N-butanol
Recovery (%)
Time (s)

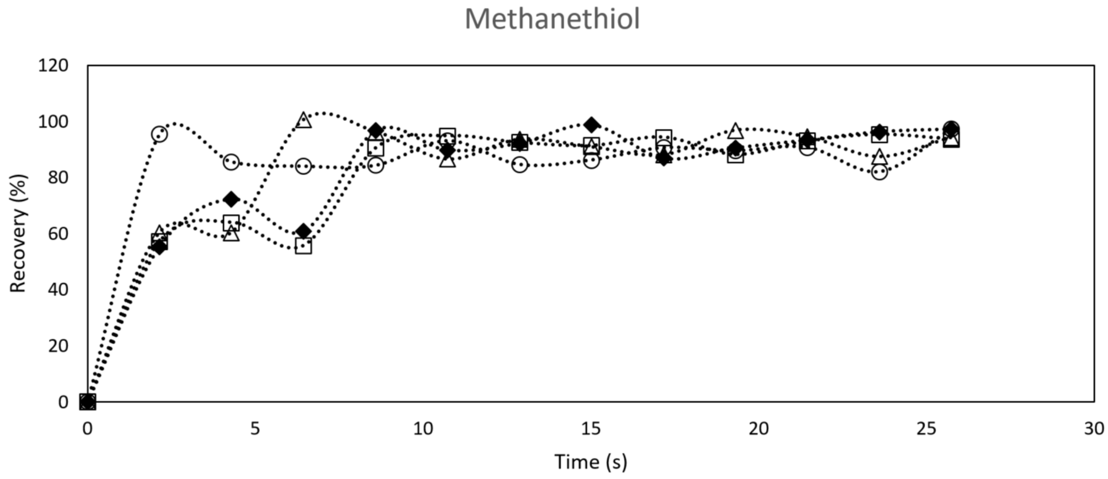
Methanethiol
Recovery (%)
Time (s)

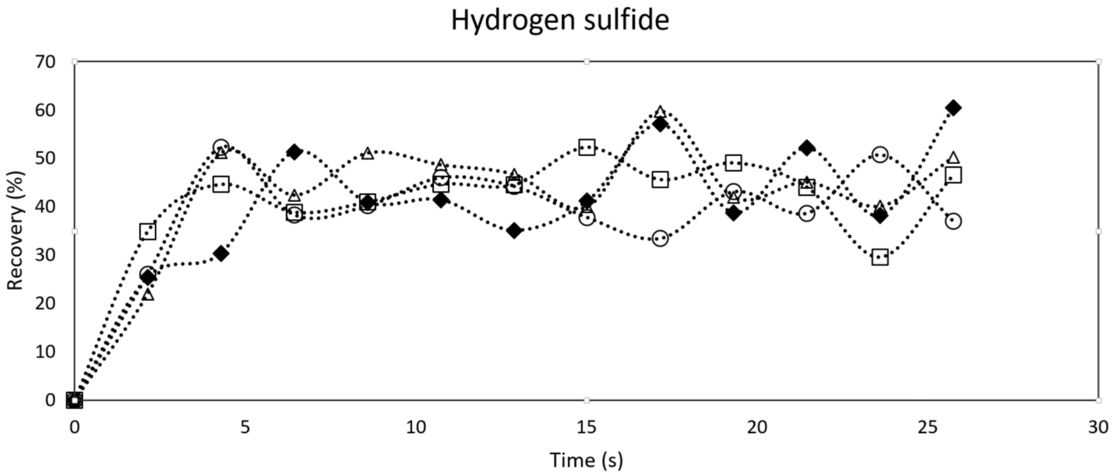
Hydrogen sulfide
Recovery (%)
Time (s)

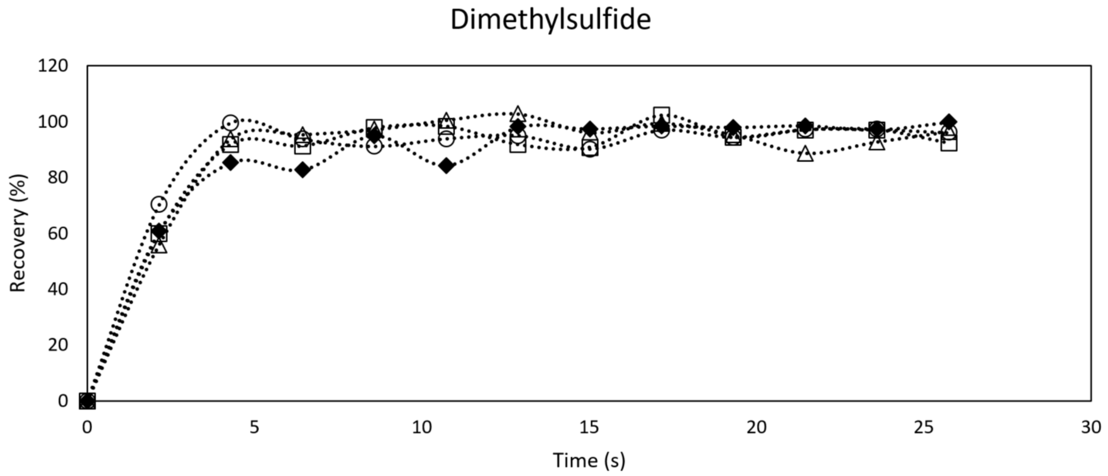

Figure S3. Recovery of odorants when flushed through tubes of different materials. Intermediate dilution (concentration levels) are presented. Dilution factor = 365. Note that, due to saturation effects) the adsorption will be increased for higher dilution factors and decreased for lower dilution factors.

···⊙··· SilcoTek ···⊡··· Stainless steel ···◆··· PTFE ···△··· PFA

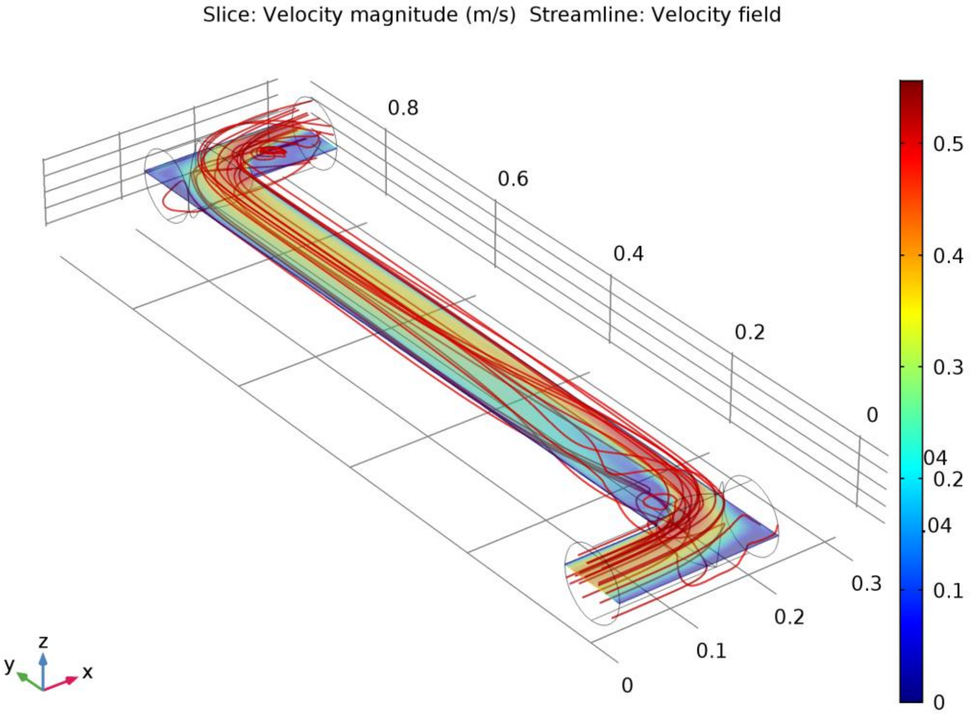

Supplemental Figure S4: Cross section of the velocity magnitude (m/s) Streamline through the velocity field showing recirculation zones at the inside of bends.
